# Iranian Journal of Basic Medical Sciences: 2019 in retrospect

**DOI:** 10.22038/IJBMS.2020.14268

**Published:** 2020-01

**Authors:** Leila Arabi, Bizhan Malaekeh-Nikouei, Ali Roohbakhsh, BiBi Sedigheh Fazly Bazzaz

**Affiliations:** 1 Nanotechnology Research Center, Pharmaceutical Technology Institute, Mashhad University of Medical Sciences, Mashhad, Iran (Assistant Editor); 2 Pharmaceutical Research Center, Pharmaceutical Technology Institute, Mashhad University of Medical Sciences, Mashhad, Iran (Assistant Editor); 3 Biotechnology Research Center, Pharmaceutical Technology Institute, Mashhad University of Medical Sciences, Mashhad, Iran (Editor–in–Chief)

The Iranian Journal of Basic Medical Sciences (IJBMS), as one of the 24 scientific journals of Mashhad University of Medical Sciences (MUMS) (1), is an internationally prestigious journal and proudly the leading Iranian multidisciplinary journal in the medical field. IJBMS is encouraging and exploring new ideas of current trends in various fields, including anatomical sciences, biochemistry, genetics, immunology, microbiology, pathology, pharmacology, pharmaceutical sciences, and physiology, by publishing papers containing pure knowledge. 

**Figure 1:**
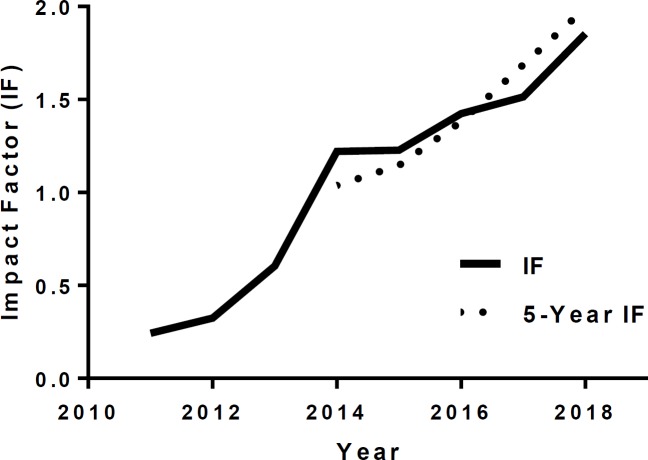
Upward trend of Impact Factor of IJBMS between 2011–2019

The quality of the journal has been recognized by the Thomson Reuters Impact Factor (IF) with its two-decade upward trend (2), which has reached the current IF of **1.85** (Figure 1). 

**Figure 2:**
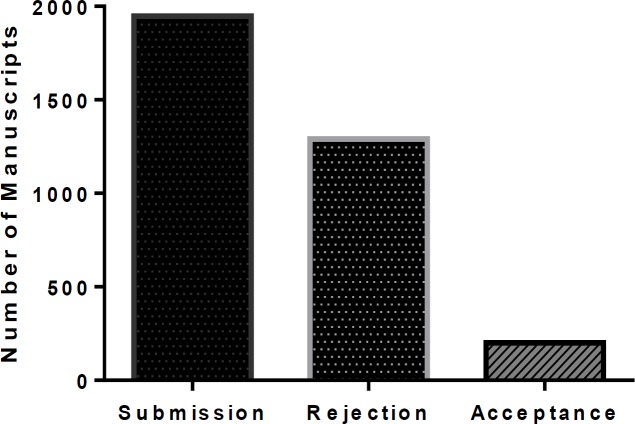
Analysis of submitted, rejected, and accepted manuscripts in 2019

The number of submitted manuscripts to IJBMS has increased during 2019 and is expected to rise further in the next year, in line with the progress of its profile and influence in the scientific community over the past few years. Just to put it into perspective, we have compared the number of submitted, rejected, and accepted manuscripts in 2019 (Figure 2).

Nevertheless, we take notice of the fact that as scientific research has expanded worldwide during the recent decades, growing concerns regarding the aim and quality of research projects have been raised too. A paper published in The Lancet in 2009 estimated that about 85% of research investment—equating to tens of billions of dollars yearly—is wasted (3)**.** Based on the meta-analysis of the primary outcome of Cochrane reviews published in the British Medical Journal (BMJ), a significant burden of the so-called “wasted research” is related to inadequate methods that could be avoided to a considerable extent by simple and inexpensive adjustments (4). Therefore, we rigorously pay attention to the methodologic and scientific soundness of our published papers. Ordinarily, the submitted manuscripts are initially examined by the editor-in-chief or the associate editors in order to verify that the major requirements have been met. These requirements include novelty of the study, fitting the scope of the journal, and complete conformity with the author guidelines and the ethical principles. Provided the main criteria are fulfilled, the review process would be started, during which two or more referees review the manuscript. Based on their careful assessment and evaluation, the decision would be made by the editorial team. 

We would like to acknowledge our reviewers' time and effort and express our genuine gratitude for their thoughtful comments and recommendations and insightful guidance.

We have strived in recent years to follow the ethics and policies of the Committee on Publication Ethics (COPE). For instance, we have dealt with two special cases during 2018, which were successfully addressed by our editorial team in full accordance with the COPE guidelines.

In addition, in order to meet the quality standards for COPE membership, we expanded our editorial board members and invited international members from the United States, Singapore, and Turkey.

Furthermore, we improved and clarified the "Guide for authors" specifically in terms of ethical concerns to develop the transparency and standards of IJBMS further.

In addition, we have also tried hard to enhance the scientific-writing standards of the published papers to motivate readers and grab their interests.

In the end, we would also like to take this opportunity to wish our readers and colleagues a Very Happy New Year. We aim at improving the quality of our journal and enjoy yet more credibility in the next year.
